# The Impact of Household Economics on Short-Term Outcomes in a Posterior Fossa Tumor Population

**DOI:** 10.7759/cureus.8968

**Published:** 2020-07-02

**Authors:** Rachel Blue, Ryan Dimentberg, Donald K Detchou, Gregory Glauser, Kaitlyn Shultz, Scott McClintock, Neil R Malhotra

**Affiliations:** 1 Neurosurgery, Perelman School of Medicine, University of Pennsylvania, Philadelphia, USA; 2 Statistics, West Chester University of Pennsylvania, West Chester, USA

**Keywords:** brain tumor, outcome disparities, posterior fossa, readmissions, socioeconomics

## Abstract

Background

Disparities exist in medical care and may result in avoidable negative clinical care outcomes for those affected. There remains a paucity in the literature regarding the impact of economic disparities on neurosurgical outcomes.

Methods

A total of 283 consecutive posterior fossa brain tumor resections, excluding cerebellopontine angle tumors, over a six-year period (June 07, 2013, to April 29, 2019) at a single, multihospital academic medical center were analyzed retrospectively. Outcomes evaluated included 30-day readmission and mortality, emergency department (ED) evaluation, unplanned return to surgery within 30 days, and return to surgery after index admission within 30 days. The population was divided into quartiles based on median household income, and univariate analysis was conducted between the lowest (Q1) and highest (Q4) socioeconomic quartiles, with significance set at a p < 0.05. Stepwise regression was conducted to determine the correlations among study variables and identify confounding factors.

Results

Whole population univariate analysis demonstrated lower socioeconomic status (SES) to be correlated with increased mortality within 30 post-operative days and increased return to surgery after index admission. No significant difference was found with regard to 30-day readmission, ED evaluation, unplanned reoperation, or return to surgery after index admission. Decreasing, but not significant, mortality was demonstrated between Q1 and Q4 socioeconomic quartiles.

Conclusions

This study suggests that low SES, when defined by household income, correlates with increased mortality within 30 days and an increased need for return to surgery within 30 days. There may be an opportunity for hospitals and care providers to use SES to proactively identify high-risk patients and test the impact of supports in the post-operative setting.

## Introduction

Social disparities impact healthcare throughout the medical experience, including access to care, provider of care, care provided, and outcome of care once access has been attained [1]. In the American healthcare system, there are many sources of disparity that have been demonstrated to impact care, including economics, race, gender, and ethnicity [2]. With the recent shift toward value-based models of care delivery, the focus on the social determinants of health (SDOH) has appropriately intensified, given that disparities may result in avoidable negative clinical outcomes as well as increased healthcare costs [3]. SDOH encompass all factors external to the immediate medical setting that impact a patient’s health and increase risk for adverse outcomes [4]. Such factors include, but are not limited to, level of education, literacy, occupation, income, nutrition, and social environment, in addition to those listed above [3]. This shifting framework for thinking about health as it relates to social disparities is reflected in the recent collaboration between the American Medical Association and UnitedHealthcare to create 23 new ICD-10 (International Classification of Diseases, 10th Revision) codes related to SDOH [5].

Studies investigating the effect of SDOH on healthcare have gained traction over the past decade and aided in bringing public and academic awareness of this issue [6-9]. Currently, there exists extensive literature correlating race [9-10], socioeconomic status (SES) [11-14], and insurance status [8] with increasing disparities for several disease categories and medical services [1]. Importantly, these documented inequities consistently impact post-surgical morbidity and mortality, skewing patient care and outcomes due to socially and culturally defined factors.

To date, there have been numerous investigations studying the effect of socioeconomics on access to care [11-14]. For example, Mukherjee et al. tangentially relate socioeconomics to access to care, implicating admission to high-quality surgical centers by wealthier patients as a proxy for economic-based inequities [15]. In contrast, the mechanisms underlying economic disparities after access has been attained are poorly defined and not well understood. Evidence from the Netherlands, which has been credited as the most equally accessible healthcare system in the world, notes increased post-operative mortality in low SES patients [16]. Additionally, a study in three Italian cities suggested that economically disadvantaged patients were less likely to be treated in concordance with clinical guidelines and had correspondingly worse prognoses [17]. However, despite these global insights, the influence of economics on outcomes for those overcoming barriers to access, particularly in the United States, is in need of further investigation.

This study examines the effect of median household income (MHI) on short-term outcomes following posterior fossa brain tumor resection (inclusive of brainstem lesions), excluding cerebellopontine angle (CPA) tumors. CPA tumors were excluded as these tend to be unique with regard to pathology and complication rate relative to other tumors of the posterior fossa [18]. Posterior fossa brain tumor resection is of particular interest due to the high rate of post-operative complications in comparison to supratentorial tumor resection [18]. Furthermore, the potential complications following posterior fossa resection are unique due to critically important structures in the region and related neurological deficits secondary to injury, including brain stem distortion, cranial nerve palsies, ataxia, and loss of respiration [18].

Low familial socioeconomic background correlates with increased risk of neurological deficits and worse long-term survival [19] after posterior fossa tumor resection. Despite pre- and peri-operative measures implemented to minimize complications, there is a continued need to systematically reduce morbidity and mortality in the posterior fossa brain tumor resection population. Given the complex interactions between SDOH, this study attempts to isolate for SES by mediating the effects of race and gender, among other clinically relevant covariates, to determine the relationship between income and outcomes following posterior fossa tumor resection.

## Materials and methods

Sample selection

**Figure 1:**
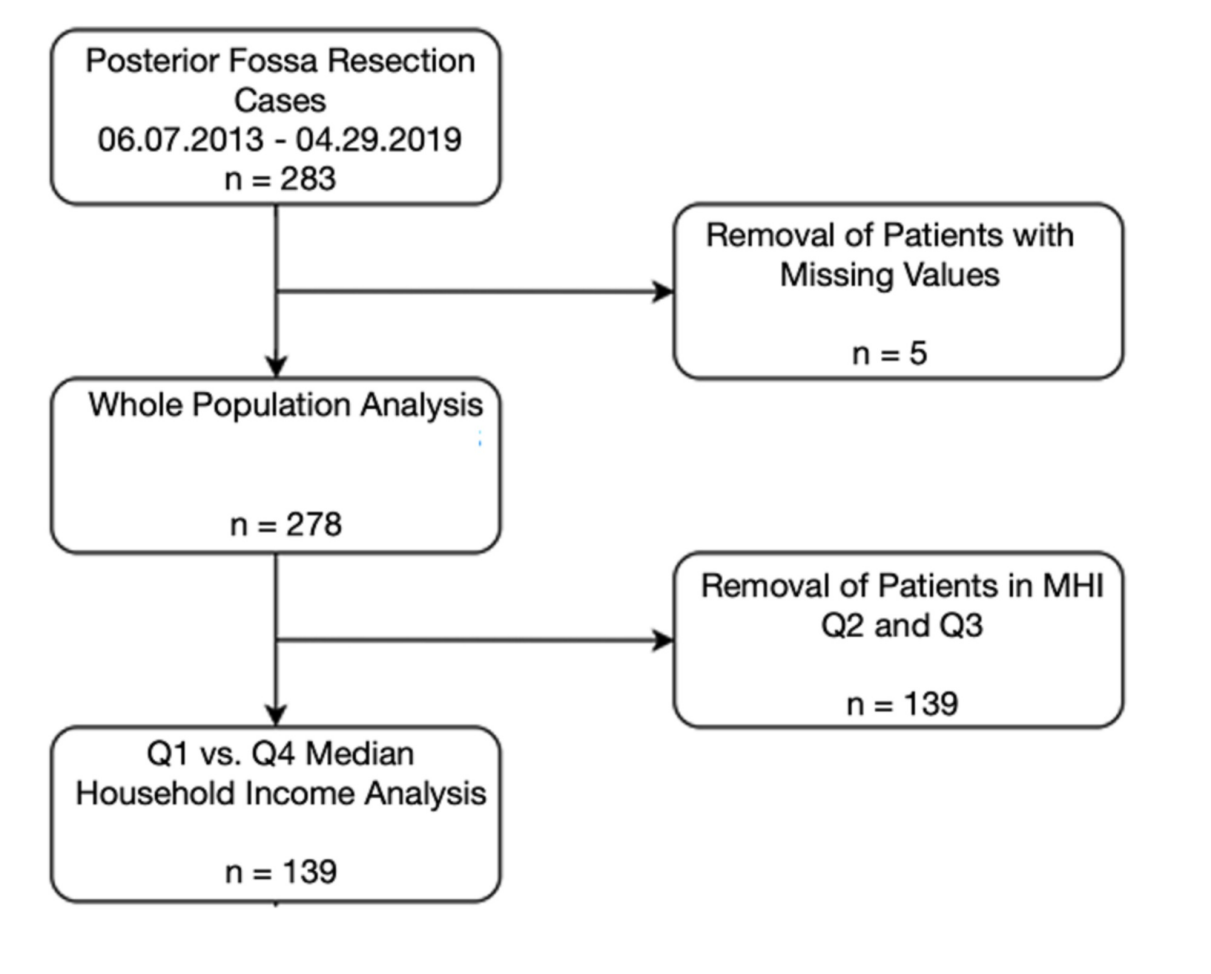
Flow Chart of Patient Selection

In this Institutional Review Board (IRB) approved study, 283 consecutive patients undergoing posterior fossa tumor resection at a multi-hospital 1,659-bed university health system over six years (June 07, 2013, to April 29, 2019) were enrolled retrospectively (Figure 1). A waiver of informed consent was granted by the IRB as this study was considered to be of minimal risk to patients. Key data were acquired using the EpiLog tool, a non-proprietary data acquisition system created by the senior author of this study. It was built and layered on top of the existing electronic health record architecture to facilitate charting, workflow, quality improvement, and cost reduction initiatives.

Data collection

Patient and outcome data for this study were extracted from EpiLog and pushed into defined spreadsheets. Patient characteristics including MHI age, gender, race, American Society of Anesthesiologists (ASA) score [20], smoking status, body mass index (BMI), Charlson Comorbidity Index (CCI) [21] and underlying covariates, duration of surgery, total cost, history of prior resection, and level of education were recorded (Table 1). Outcomes assessed were unplanned readmissions, emergency department (ED) evaluation, unplanned return to surgery, return to surgery after index admission, and mortality within 30 post-operative days.

**Table 1:** Patient Demographics and Baseline Characteristics Q1, quartile 1; Q4, quartile 4; SD, standard deviation; CCI, Charlson Comorbidity Index; AIDS, acquired immunodeficiency syndrome; BMI, body mass index; ASA, American Society of Anesthesiologists

Variable	Median Household Income Q1 (N = 70)	Median Household Income Q4 (N = 69)	Standardized Difference
Age, mean (SD)	58.16 (13.46)	52.42 (15.76)	-0.3914
Length of stay in days, mean (SD)	10.98 (8.43)	6.88 (6.48)	-0.5447
Gender			
Female, n (%)	31 (44.29)	38 (55.07)	-0.217
Male, n (%)	39 (56.31)	32 (44.93)	-0.217
Total cost of surgical implants and supplies in US dollars, mean (SD)	2505.44 (1622.84)	3092.22 (3278.82)	0.2268
Duration of surgery in minutes, mean (SD)	245.22 (123.07)	225.65 (90.26)	-0.1813
CCI score, mean (SD) (components: myocardial infarction, congestive heart failure, peripheral vascular disease, transient ischemic attack, dementia, chronic obstructive pulmonary disorder, connective tissue disease, peptic ulcer disease, liver disease, diabetes, hemiplegia, chronic kidney disease, solid tumor, leukemia, lymphoma, AIDS)	5.93 (3.85)	4.55 (3.73)	-0.3636
Number of surgical interventions within 90 days prior to surgery, n (%)			
0	61 (87.14)	67 (97.10)	0.3822
1	8 (11.43)	2 (2.90)	
2	1 (1.43)	0 (0)	
BMI, mean (SD)	26.75 (6.80)	26.44 (5.96)	-0.0484
ASA grade, n (%)			
1	0 (0)	0 (0)	0.3221
2	14 (20.00)	23 (33.33)	
3	52 (74.29)	44 (63.77)	
4	4 (5.71)	2 (2.90)	
Tobacco use (within past 12 months), n (%)			
Yes	16 (22.86)	5 (7.25)	0.4617
No	45 (64.29)	56 (81.16)	
Unknown	9 (12.86)	8 (11.59)	
Race, n (%)			
Asian	1 (1.43)	6 (8.70)	1.0544
Black	28 (40.00)	2 (2.90)	
White	37 (52.86)	55 (78.57)	
Other	4 (5.72)	6 (8.70)	

Statistical analysis

Univariate analysis was performed with respect to MHI, with significance set as p < 0.05. Patients were divided into quartiles based on MHI, and an additional univariate analysis was conducted between the lowest (Q1) and highest (Q4) socioeconomic quartiles, with a significance set as p < 0.05. Stepwise regression was conducted to identify confounding variables by analyzing the effect of individual variables known to impact outcomes on the observed correlation. This was conducted for all variables.

## Results

Patient characteristics

The sample included all consecutive patients undergoing posterior fossa tumor resection, excluding CPA tumors, over the course of six calendar years (n = 283). Four patients with missing values and one patient with a BMI less than 10 were excluded from analysis. For the resulting patient cohort, the mean age was 54.7 years (Table 1). Around 11.5% of the studied patients used tobacco within the 12 months prior to surgery. The mean length of stay was 201 hours, and the cohort comprised 54% females.

Patient outcomes

**Figure 2:**
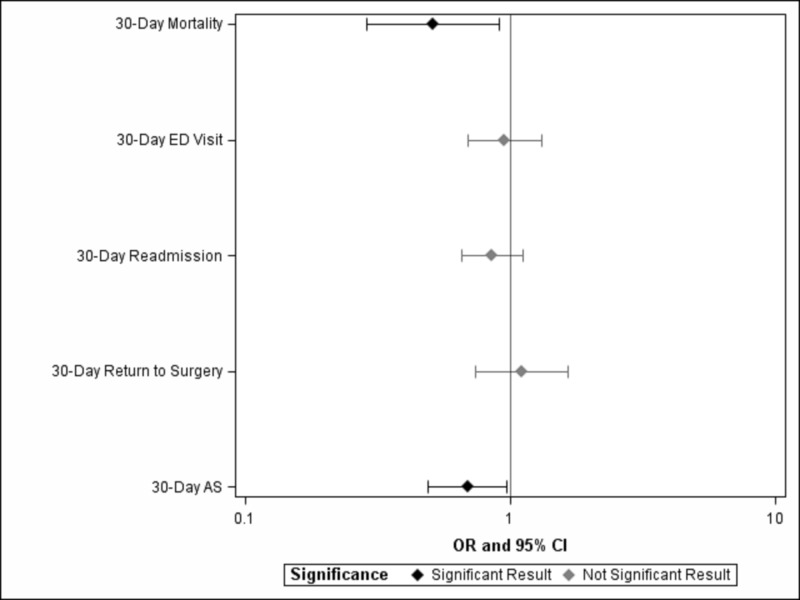
Odds of Adverse Outcome and Complication with Increasing Median Household Income across the Whole Study Population 30-day AS refers to return to surgery after index admission within 30 days. Significance is set at p < 0.05. OR, odds ratio; CI, confidence interval

**Figure 3:**
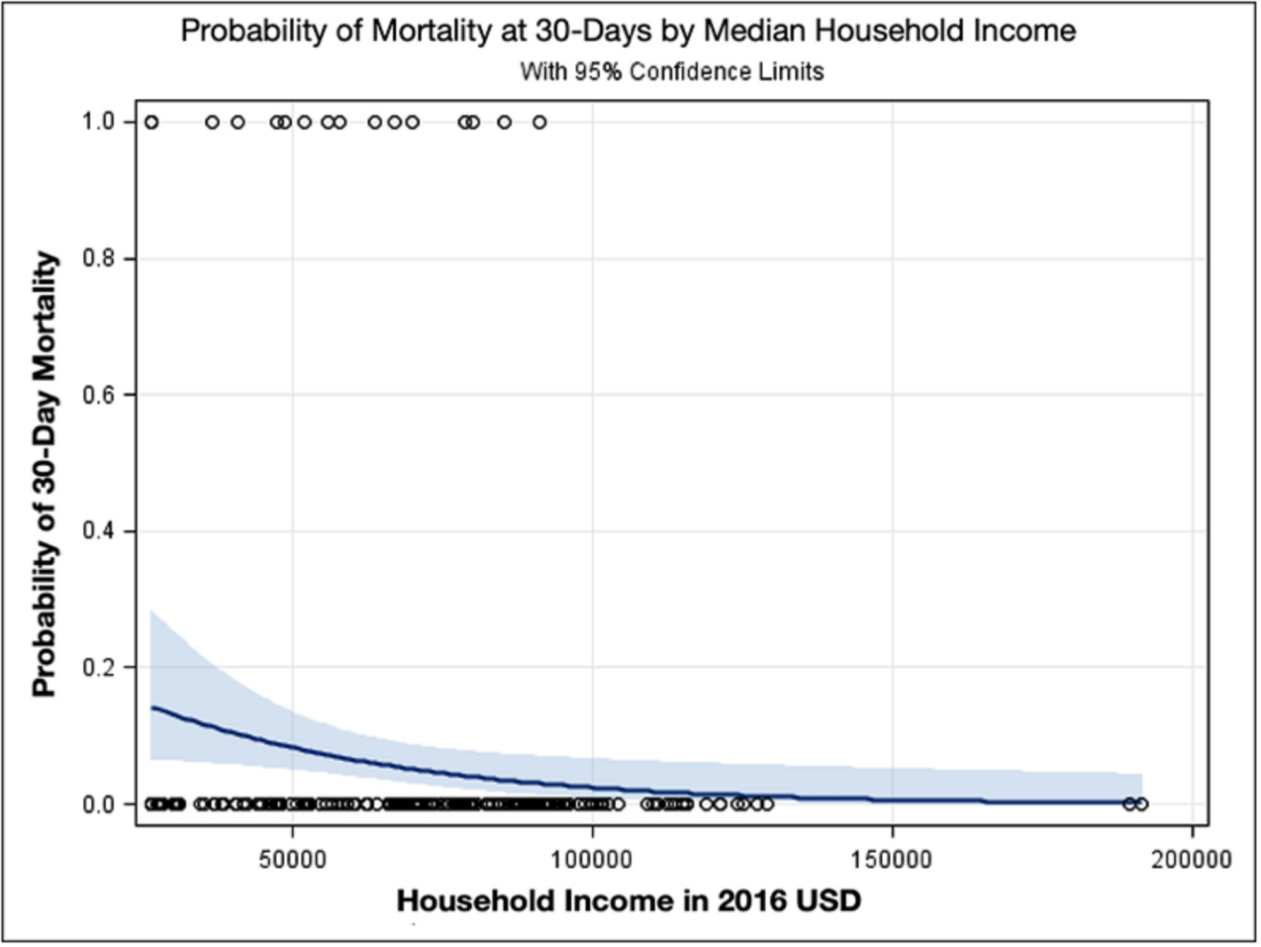
Probability of Mortality at 30 Days by Increasing Median Household Income

**Figure 4:**
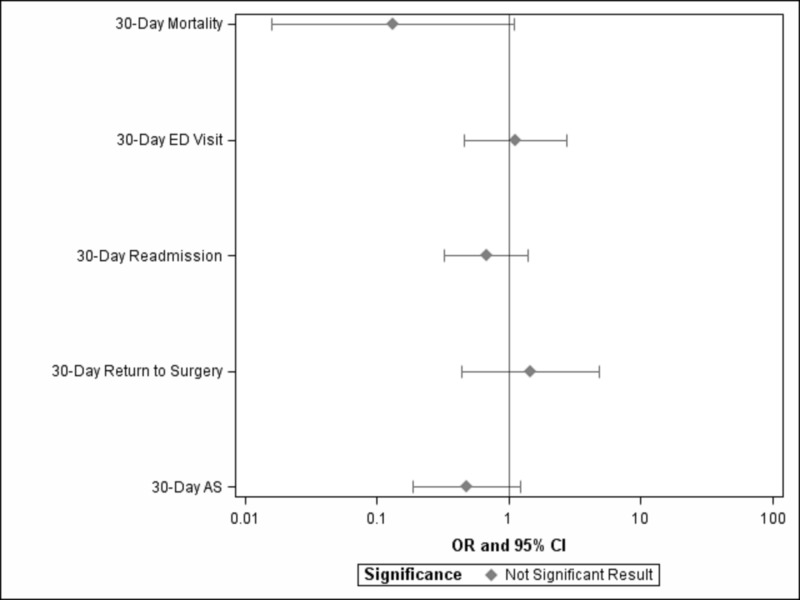
Odds of Adverse Outcomes and Complications between Patients in the Highest (Q4) and the Lowest (Q1) Socioeconomic Quartiles 30-day AS refers to return to surgery after index admission within 30 days. Significance is set at p < 0.05 OR, odds ratio; CI, confidence interval; Q1, quartile 1; Q4, quartile 4

Whole population univariate analysis demonstrated lower SES to be significantly correlated with increased mortality within 30 post-operative days (p = 0.03; OR = 0.69; 95% CI = 0.49-0.97) and increased return to surgery after index admission (p = 0.02; OR = 0.51; 95% CI = 0.29-0.91) (Figures 2, 3). No significant difference was found with regard to 30-day readmission (p = 0.24), ED evaluation (p = 0.75), or unplanned reoperation (p = 0.63). Between Q1 and Q4 socioeconomic quartiles, decreasing but not significant mortality was observed (p = 0.06; OR = 0.13; 95% CI = 0.02-1.12) (Table 2, Figures 3, 4). Additionally, no significant difference was found for 30-day readmission (0.29), ED evaluation (p = 0.79), unplanned reoperation (p = 0.53), or return to surgery after index admission between Q1 and Q4.

**Table 2:** Q1 and Q4 Patient Outcomes and Complications Q1, quartile 1; Q4, quartile 4

Outcome/Complication	Patient Groups		
	MHI Q1, n (%)	MHI Q4, n (%)	
			30-day mortality	7 (10.00)	1 (1.45)	
				p = 0.06		
OR = 0.13 (95% CI: 0.02-1.11)			
30-day readmission	24 (34.29)	18 (26.09)	
	p=0.29		
	OR = 1.48 (95% CI: 0.71-3.07)		
30-day ED evaluation	11 (15.71)	12 (17.39)	
	p = 0.79		
	OR = 0.89 (95% CI: 0.36-2.17)		
30-day reoperation	5 (7.14)	7 (10.14)	
	p = 0.53		
	OR = 0.68 (95% CI: 0.21-2.26)		
30-day return to surgery after index admission	15 (21.43)	8 (11.59)	
	p = 0.12		
	OR = 0.48 (95% CI: 0.19-1.22)		

Stepwise regression

Thirty-day readmission and 30-day ED evaluation were significantly predicted by a history of surgery within 90 days and BMI. Additionally, 30-day unplanned return to surgery was associated with BMI, and unplanned return to surgery after index admission was associated with duration of surgery and BMI. Furthermore, mortality within 30 days was associated with ASA grade. Notably, race, gender, insurance status, age, cost of surgery, and the remaining characteristics demonstrated little effect on the observed correlation for any of the primary or secondary endpoints.

## Discussion

In this study, univariate regression of 278 consecutive patients undergoing posterior fossa tumor resection, excluding CPA tumors, demonstrated that increasing household income correlates with decreased mortality within 30 days and decreased return to surgery after index admission within 30 days. Furthermore, univariate analysis between the lowest and highest socioeconomic quartiles demonstrated a decreasing but not significant difference in mortality. In both analyses, no significant difference was observed for 30-day readmission, ED evaluation, or unplanned reoperation.

The effect of SES on surgical outcomes is a topic of increasing interest for the surgical community. Unfortunately, the interrelation between SES and various other SDOH makes it difficult to consider in isolation [11-14]. SES has been shown to impact access to surgical care, which has been demonstrated to contribute to poor outcomes, but the effect beyond access to care remains unclear. This study, in congruence with prior work in the literature, evaluates outcomes after access has been attained in order to more directly measure the influence of economics on the outcomes studied. This study makes no claims as to the effect of economics on access to care.

Additionally, the use of univariate and stepwise logistic regression enables confounding covariates to be mediated. As such, the described relationship corrects for patient and procedure characteristics known to substantially impact outcomes. This is particularly significant when evaluating SDOH due to the complex interactions between multiple factors. The criteria selected for this study were based on supporting literature indicating the relationship of each characteristic with post-surgical outcomes. Numerous studies have demonstrated race [22], smoking status [23], BMI [24-25], CCI [26-28], duration of surgery [29], and ASA score [30] to independently predict morbidity following surgery. This study is of further value to the literature as the influence of SES on outcomes has never been independently evaluated in a posterior fossa brain tumor population. While previous studies have suggested a relationship between socioeconomics and outcomes following posterior fossa tumor resection, they do not systematically mediate for the above covariates, introducing potentially significant confounding bias. Notably, regression analysis demonstrates that the present findings are unaffected by race, gender, insurance status, cost of surgery, and age, amongst other factors, suggesting adequate control of confounding variables. This demonstrates robust control of other SDOH and successful isolation of SES.

Historically, surgeons were hesitant to perform surgeries to treat posterior fossa disorders because this brain region is highly sensitive to manipulation, with potential for neurologically devastating complications. A review of complications following surgeries localized to that area found an overall complication rate of 31.8%, affecting 159 of 500 patients assessed [18]. Such complications include cerebrospinal fluid leaks (13%), meningitis (9.2%), wound infection (7%), and cranial nerve palsies (4.8%). Additional intraoperative complications have been found to result in brain stem compression, loss of respiration, and sudden death [18]. Various pre- and peri-operative measures have been described, which reduce overall morbidity and mortality, including quality interval neurological assessments, imaging studies, aseptic technique, meticulous microsurgical dissection, watertight dural and wound closure, and prophylactic measures [18]. However, novel strategies to improve post-operative outcomes continue to be of great need.

As hospitals and health systems move toward data-driven medicine, the present findings suggest that SES may be a useful metric for predicting patients with increased risk of mortality following posterior fossa tumor resection. It is worth considering that outcome differences across SES status are potentially attributable, in part, to when patients sought treatment, as patients of lower SES may be less inclined to seek medical care early when tumors are more easily resected. Leveraging such insights may enable additional resources to be allocated to high-risk patients (transportation coordination, meal assistance, automatic check-in messages, etc.) and facilitate additional pre-operative optimization efforts and diligent post-operative follow-up scheduling. As more studies validate the present findings, it is increasingly important for departments to proactively identify at-risk patients in order to improve outcomes. The electronic health record makes it increasingly easy to identify and follow these patients, providing a powerful means of improving quality and reducing costs with minimal workflow interruption.

There are several limitations to this study. One such limitation is that this study is retrospective; therefore, potential sampling bias and data recording inaccuracies may exist. Furthermore, the study assesses readmissions and ED evaluation based on electronic health record data from the university hospital system in which the study was conducted, assuming that this system would be the recipient of all such events. As such, there is a potential for underreporting of true readmission and ED evaluation rates. Nonetheless, study patients had significant follow-up (median of 482 days) during which discrete data was captured in reference to all healthcare received. Furthermore, any remaining discrepancies would likely be consistent across patients of multiple income-levels and therefore does not significantly affect the internal validity of the study. Secondly, as with all retrospective studies, the influence of each covariate could not be completely controlled for despite being incorporated into the model by stepwise regression. As demonstrated, return to surgery after index admission within 30 days was associated with the duration of surgery and BMI. Furthermore, there is an association between 30-day mortality and ASA grade, which may have contributed to the observed trend. Future studies prospectively analyzing these outcomes in a larger, matched population is required to further validate our findings.

To expand on this study, future research should investigate the effects of other SDOH on post-operative outcomes following posterior fossa brain tumor resection in order to better isolate the driving cause of disparity. Additionally, further studies exploring the influence of socioeconomics on morbidity and mortality at mid- and long-term follow-up may further elucidate the need to include SES into care protocols to predict and avoid subsequent complications.

## Conclusions

This study demonstrates that economics affects short-term outcomes following posterior fossa brain tumor resection. Across the whole population, low SES is significantly associated with increased return to surgery after index admission and increased mortality at 30 days. Further investigation at mid- and long-term follow-up are required to expand the utility of the present findings. The findings of this study are significant given the importance of eliminating disparities in healthcare, and the growing interest in the role of SDOH in medicine and surgery. Furthermore, this study suggests that there are opportunities for correcting disparities by proactively targeting low SES patients with additional interventions, which the authors hope to investigate in future studies.
